# HOXB13-Mediated Lipid Metabolism Regulates Prostate Cancer Metastasis

**DOI:** 10.17161/sjm.v1i1.23157

**Published:** 2024-12

**Authors:** Jianhua Xiong

**Affiliations:** 1 Department of Urology, Emory University School of Medicine, Atlanta, GA, USA;; 2 Winship Cancer Institute of Emory University, Atlanta, GA, USA;

**Keywords:** androgen receptor, HDAC3, HOXB13, lipogenesis, prostate cancer

## Abstract

The molecular mechanisms driving increased lipogenesis and lipid accumulation, key features of prostate cancer (PCa), remain largely unexplored. Recently, Lu and colleagues identified a novel role for the homeodomain transcription factor HOXB13 in regulating lipid metabolism and PCa progression, independent of its traditional involvement in androgen receptor (AR) signaling. Their study demonstrates how HOXB13 modulates PCa metastasis through epigenetic reprogramming, specifically by interacting with histone deacetylase 3 (HDAC3). These findings not only deepen our understanding of the molecular processes underlying PCa metastasis but also suggest lipid metabolism as a promising therapeutic target for castration-resistant and HOXB13-low PCa.

Lipids are essential biomolecules with diverse roles, including serving as energy reservoirs, building blocks of cellular structures, and key signaling molecules [1]. Defined by their high solubility in nonpolar solvents and limited solubility in polar solvents (e.g., water), lipids can be broadly categorized into two main groups: sterols, such as cholesterol, and fatty acid-based lipids. The latter group includes fatty acids, triacylglycerols, phospholipids, and sphingolipids. The significance of lipid research is underscored by its recognition with three Nobel Prizes [1,2]. Most cells can convert carbohydrates into fatty acids, which are commonly stored as neutral lipids within lipid droplets. Emerging evidence highlights the critical role of lipogenesis not only in metabolic tissues, where it maintains systemic energy homeostasis but also in other systems, including the urological system, where it supports essential processes such as cell function and motility [1,2]. Any disruption in lipogenesis—whether excessive or insufficient—can destabilize lipid homeostasis, potentially leading to pathological conditions such as prostate cancer (PCa) [3,4].

Dysregulated lipid metabolism is a hallmark of PCa development and progression. Lipid droplet accumulation in aggressive tumors, metastatic lesions, and circulating prostate tumor cells underscores its role in disease aggressiveness [3–8]. To maintain energy balance, enzymes involved in lipogenesis are tightly regulated through transcriptional and post-translational modifications. One such enzyme, fatty acid synthase (FASN), is upregulated in PCa tissue and correlates with Gleason scores, making it a potential biomarker [9]. FASN plays a key role in de novo lipogenesis by synthesizing palmitic acid and activating sterol regulatory element-binding proteins (SREBPs), which govern the expression of enzymes involved in sterol biosynthesis [6–9]. The androgen receptor (AR), a ligand-activated nuclear transcription factor and a central driver of PCa, also significantly impacts lipid metabolism by modulating the expression of enzymes related to lipid synthesis, uptake, and utilization [10–12]. Androgens promote lipid accumulation in PCa cells, and the reactivation of AR-driven lipid biosynthesis has been linked to the progression of metastatic castration-resistant prostate cancer (CRPC) [13]. Additionally, factors such as PTEN loss, MAPK pathway activation, and the nuclear pyruvate dehydrogenase complex contribute to the complexity of lipid metabolism in PCa progression [14–16]. However, despite these insights, the precise molecular mechanisms driving increased lipogenesis and lipid accumulation in PCa remain poorly understood.

A recent study by Lu *et al*. revealed a previously unrecognized function of HOXB13, a transcription factor from the homeobox (HOX) family, in controlling lipid metabolism in PCa, distinct from its traditional role in AR signaling [17]. The authors demonstrate that HOXB13 suppresses de novo lipogenesis by recruiting histone deacetylase 3 (HDAC3) to lipogenic enhancers, leading to histone deacetylation and the downregulation of key lipogenic genes, such as FASN (Figure 1). This epigenetic mechanism is significant because it links chromatin remodeling with metabolic regulation-two processes increasingly recognized as central to cancer progression. Prior to this study, the role of HOXB13 outside of AR signaling had been largely unexplored. HOXB13 is predominantly expressed in the adult prostate gland [18,19]. In PCa, HOXB13 regulates AR activity by modulating AR binding and transcriptional programs, influencing both the initiation and progression of the disease. This regulation occurs through its interactions with other key factors, such as FOXA1, which helps redirect AR to PCa-specific chromatin targets. HOXB13’s effects on AR binding are context-dependent, varying based on the cellular environment and the presence of specific co-factors [20–23]. Although HOXB13 is most recognized for its involvement in androgen-driven PCa progression [20–23], mutations in HOXB13, especially the G84E variant, have been associated with early-onset familial forms of PCa [24]. Lu *et al*. reveal that wild-type HOXB13, unlike its G84E mutant variant, suppresses lipogenic pathways by facilitating histone deacetylation through the recruitment of histone deacetylase 3 (HDAC3), a crucial step in suppressing de novo lipogenesis (Figure 1A). In contrast, the G84E mutation impairs HOXB13’s ability to interact with HDAC3, disrupting its role in inhibiting lipogenesis (Figure 1B). This disruption offers a molecular explanation for the aberrant lipid accumulation observed in PCa cells harboring the G84E mutation.

This work represents a significant advancement in understanding the clinical relevance of HOXB13 in metastatic CRPC. In approximately 30% of metastatic CRPC tumors, HOXB13 is hypermethylated and downregulated. The loss of HOXB13 or the presence of the G84E mutation leads to lipid accumulation in prostate cancer cells, which, in turn, enhances their motility and metastatic potential (Figure 1B). This finding underscores the growing recognition of lipid metabolism as a crucial driver of tumor aggressiveness and metastasis. The authors also show that pharmacological inhibition of FASN, a key target of HOXB13 regulation, can mitigate the adverse effects of HOXB13 loss, offering a promising therapeutic strategy for patients with low or mutated HOXB13 expression. One of the study’s key strengths is its translational potential. By identifying HOXB13 as a regulator of lipid metabolism, the research provides a compelling rationale for targeting the lipogenic pathway in HOXB13-deficient or low-expression prostate cancer. Given that FASN inhibitors are already being developed for other cancers [25–27], this study paves the way for their potential application in prostate cancer, especially in cases where conventional androgen-targeted therapies have been ineffective. Furthermore, the discovery of HOXB13’s role in lipid metabolism opens avenues to explore additional metabolic pathways it may influence, broadening our understanding of its role in cancer biology.

Despite the promising findings, several important questions remain to be addressed. One key area is understanding the precise upstream signals that regulate HOXB13’s interaction with HDAC3 and its recruitment to lipogenic enhancers. This molecular mechanism is not yet fully understood, and uncovering these regulatory pathways could provide valuable insights into how lipid metabolism is controlled in prostate cancer. Additionally, while the study demonstrates the efficacy of FASN inhibition in preclinical models, further research is needed to explore the broader therapeutic potential of targeting lipid metabolism in human clinical trials. Currently, FASN inhibitors are emerging as promising therapeutic agents in cancer treatment, with several preclinical and clinical studies underway. These inhibitors, such as TVB-2640 and orlistat, are being investigated for their ability to modulate lipid metabolism and potentially counteract metabolic reprogramming in cancer. Orlistat, in particular, has shown potential to inhibit tumor growth across various cancer models [25]. TVB-2640, in combination with bevacizumab, has shown promising results in Phase II clinical trial for patients with first-relapsed high-grade astrocytoma [26]. Additionally, the broader potential of FASN inhibitors in oncology has been discussed, suggesting their utility across different cancer types, including breast and prostate cancer [27]. However, a major challenge lies in the potential for resistance to metabolic therapies, particularly in tumors with aberrant metabolic reprogramming [28]. This resistance could undermine the effectiveness of lipid-targeting treatments over time, highlighting the importance of understanding the mechanisms behind metabolic reprogramming and its role in therapy resistance.

In addition to the anabolic pathway of lipogenesis, the catabolic process of fatty acid oxidation-which degrades long-chain fatty acids primarily in mitochondria-plays a critical role in PCa bioenergy metabolism [29,30]. Fatty acid oxidation has been shown to regulate cell fate and function in cells within the tumor microenvironment, such as endothelial cells during endothelial-to-mesenchymal transitions (EndMT), cancer epithelial cells during epithelial-to-mesenchymal transitions (EMT), and T cells involved in differentiation and survival [31–36]. The role of fatty acid oxidation in the tumor microenvironment of aggressive PCa subtypes, such as metastatic CRPC and therapy-resistant neuroendocrine prostate cancer (NEPC), remains an important area for further exploration. Additionally, short-chain fatty acids, including omega-3 and gut microbiome-derived types, are also linked to PCa progression [37–40], though the molecular mechanisms underlying these effects remain poorly understood. Recent studies suggest that acetate, a short-chain fatty acid, plays a pivotal role in regulating cell fate and function. This is mediated through its central metabolite, acetyl-CoA, which acts as a second messenger in cellular signaling, influencing both urological health and disease [41].

In conclusion, the study by Lu *et al*. significantly advances our understanding of the molecular mechanisms underlying PCa metastasis, particularly by highlighting the role of epigenetic regulation in cancer cell metabolism. By identifying HOXB13 as a suppressor of de novo lipogenesis through HDAC3-mediated epigenetic reprogramming, this research not only expands our knowledge of PCa biology but also opens new therapeutic opportunities for aggressive, castration-resistant forms of the disease. As metabolic therapies continue to gain traction in oncology, the insights provided by this study may prove pivotal in shaping the future of PCa treatment.

## Figures and Tables

**Figure 1.**
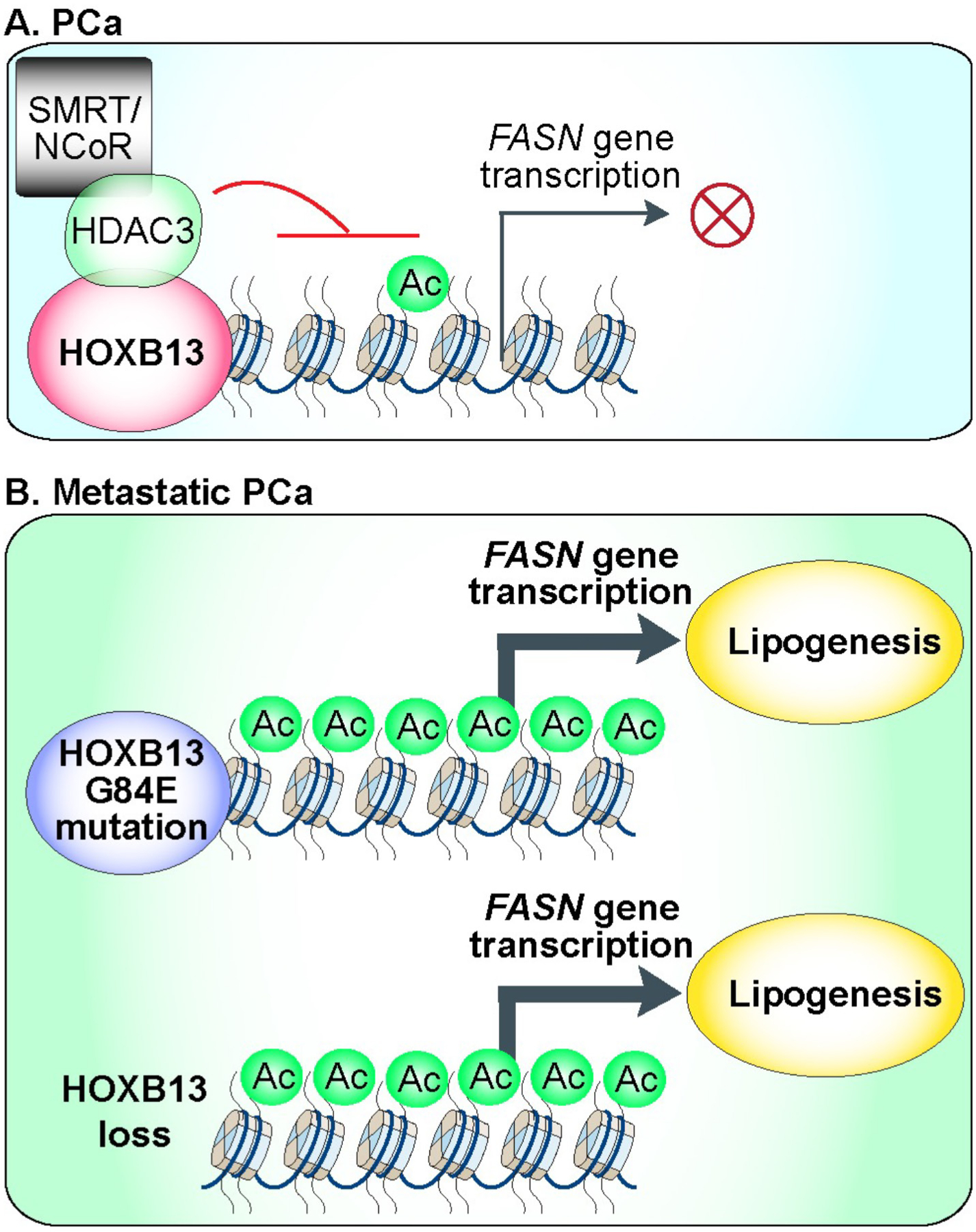
HOXB13 suppresses lipogenic signaling in prostate cancer (PCa) through the recruitment of the HDAC3/NCoR complex for histone deacetylation. **(A)** HOXB13 inhibits de novo lipogenesis in PCa by recruiting the HDAC3/NCoR complex, which deacetylates histones to repress the transcription of the fatty acid synthase (*FASN*) gene. Annotation: HDAC3 functions as the catalytic subunit of the NCoR (nuclear receptor corepressor)/SMRT (silencing mediator for retinoid and thyroid hormone receptors) complex. **(B)** The G84E mutation in HOXB13 or the loss of HOXB13 disrupts its interaction with HDAC3, leading to increased lipogenesis and promoting PCa metastasis.
